# Executive Committee Formed

**Published:** 1921-07-30

**Authors:** 


### THE HOSPITAL WEEK AND PAGEANT.

### Executive Committee Formed.

The second meeting of Hospital Secretaries was held at the National Hospital for the Paralysed and Epileptic, Queen Square, W.C., on Friday, July 15, for the purpose of further considering the proposed scheme with regard to a Hospital Week and Pageant or a Hospital Pageant only.

Mr. G. H. Hamilton, Secretary of the National Hospital for the Paralysed and Epileptic, presided, and, in opening the proceedings, reminded those present that at the previous meeting, held on May 20, it had been decided that the three Hospital Funds should be approached in order to ascertain whether they were in favour of the suggestion that there should be a pageant in aid of the hospitals. The King Edward's Hospital Fund and both the Saturday and the Sunday Hospital Funds had been written to, and before giving a very definite reply they had asked for further information with regard to the payment of expenses and the division of the proceeds, should there be any. Personally, he rather gathered that the main object of their endeavour was not so much proceeds as advertisement and calling the attention of the public to the needs of the hospitals. There were, of course, people who were attracted to help such causes because they considered they were inspired by Providence to manage schemes for raising funds in aid of hospitals. Many of those good people were not aware of what had already been tried, and wrote to hospitals suggesting schemes whereby they could overcome their financial difficulties.

### The View of the Majority.

Mr. Glenton Kerr (Queen's Hospital for Children) did not think it a fact that the public are not sufficiently appreciative at the present time of the work that the hospitals are doing, or of their needs. As a matter of fact, he believes the public consider they are hearing a little too much of hospitals so far as appeals for assistance are concerned. Therefore he is opposed to a pageant. Mr. J. M. Churchfield (St. George's Hospital) was in favour of a week and a pageant, for the former would not only advertise the hospitals, but make the scheme pay. By combining a pageant with a week a different district could be done each day, and with collectors as part of the pageant they would thus be able to tackle a whole district not only for the particular hospital in that district, but for all hospitals in London. Mr. H. H. Jennings (Chelsea Hospital) suggested they were rather putting the cart before the horse in considering whether there should be a hospital week and pageant or a pageant only before going into the question of costs; but Mr. G. Panter pointed out that it would be very difficult for the Executive Committee to formulate a scheme and ascertain the cost unless they first of all knew whether the decision was in favour of a hospital week and pageant or pageant only. Mr. H. A. Bone (Miller General Hospital) believes a pageant will link the hospitals together and give them an opportunity to co-operate in a way in which nothing else could do at the present time. Speaking from experience in South-East London, he knew that by co-operation they had obtained wonderful results.

### When to Hold a Pageant.

Mr. G. W. F. Robbins (Battersea General Hospital) was in favour of a pageant, but asked for an expression of opinion whether it should be held this year or early next spring. The Chairman said he gathered that the intention was that if a pageant were decided upon it should be held in the spring of next year. Mr. R. J. Coles (King's College) was also in favour of a pageant, considering it a means of bringing hospitals together. As far as he can see hospitals are not likely to participate in the Government grant unless they make an effort themselves. Therefore if they obtained nothing else but publicity the scheme is well worth carrying out. Mr. Glenton Kerr objected that the scheme is bound to cost a good deal of money, and in his opinion it is unnecessary. It might, however, be more feasible to have a hospital week without a pageant. If they had a week, an army of voluntary workers can do all that is necessary to prepare the public for it. The Chairman did not agree that a pageant would of necessity cost a great deal of money; and Mr. H. E. Smithers pointed out that when the pageant had been held in Edinburgh the tradespeople helped by giving quite a lot of assistance, and he thought they would do the same in London. Monsieur de Wiart (Dollis Hill) thought if a pageant were properly organised it would be a very great success, and to do any good they would need a week, or perhaps more. Mr. Panter was perfectly sure the London hospitals will never get sufficient money until they are prepared to spend a little more on letting people know what they were doing and making it easy for people to give. For instance, it had been the custom in North London to have a house-to-house collection once a year, which produced about £1,200. He had thought it desirable to have that collection made twice a year, with the result that whereas a collection once a year had produced £1,200, a collection twice a year realised £2,000 on each occasion. So that it was really worth spending money to get money. Mr. G. H. Hawkins (Samaritan Free Hospital for Women) thought there were many hospitals who would want to know something more about the cost before giving permission for their money to be used in participating in such a scheme. He thought the Committee should be given the cost of alternative schemes; the cost of a pageant only and the cost of a hospital week and pageant.

### The Alternative Proposals.

Mr. Canning (City of London Maternity Hospital) proposed that there should be a hospital week instead of a hospital pageant, because the latter, to be successful, would have to be done on such a colossal scale that the cost would be prohibitive, whilst a hospital week only could easily be boomed by some sort of concerted advertising, and in that case the cost would be small. Mr. G. H. Hawkins seconded. Mr. Panter proposed, and Mr. Coles seconded, that there should be a hospital week and a pageant.

The Chairman then put the alternative propositions to the vote and declared Mr. Panter's motion carried, eleven voting in its favour, and eight in favour of Mr. Canning's proposition.

On the motion of Mr. W. M. Wilcox (East London Hospital for Children), seconded by Mr. F. J. Jones (Kensington and Fulham General Hospital), the following were appointed an Executive Committee, with power to co-opt: Messrs. G. Panter, G. H. Hamilton, R. J. Coles, P. Inman, J. M. Churchfield, H. H. Jennings, J. McKay, H. A. Bone, representatives of the King Edward's Hospital Fund, the Hospital Sunday Fund, and the Hospital Saturday Fund, with Mr. H. E. Smithers as Honorary Secretary. It was agreed that the reference to the Committee be: To arrange a definite scheme; to ascertain the probable cost of carrying it out, and to report thereon. It was also agreed that the Executive Committee should consider the advisability of approaching the Committee of each of the London hospitals with the object of obtaining their views upon the scheme.

### The Division of Expenses.

After a further short discussion the following motion, proposed by Mr. H. A. Bone, and seconded by Mr. R. J. Coles, was unanimously carried:

"That the division of expenses of carrying out the scheme be on the basis of occupied beds, and that two thousand outpatient attendances be regarded as equal to one bed, the receipts to be divided in the same way, the figures for 1920 being taken as a basis for calculation."

A vote of thanks to the Chairman terminated the proceedings.

